# Synergistic Removal of Pb(II), Cd(II) and Cr(VI) by Chitosan-Encapsulated Phosphorus-Modified Biochar: Multi-Site Sorption and Immobilization

**DOI:** 10.3390/gels12080738

**Published:** 2026-08-18

**Authors:** Yang Feng, Min Zhou, Jiangyan Wu, Lingli Li, Haoming Chen, Lingyi Tang

**Affiliations:** 1 School of Art and Design, Xijing University, Xi’an 710123, China; 2 Culinary Science and Technology Department, Jiangsu Vocational College of Tourism, Yangzhou 225000, China; 3 School of Environmental and Biological Engineering, Nanjing University of Science and Technology, Nanjing 210094, China; 4 Department of Earth and Atmospheric Sciences, University of Alberta, Edmonton, AB T6G 2E3, Canada

**Keywords:** biochar, phosphorus modification, chitosan encapsulation, heavy metals, mineral precipitation

## Abstract

Heavy metal pollution has become a global environmental problem. Achieving efficient, stable, and sustainable immobilization of heavy metals by phosphorus (P)-modified biochar remains challenging because of the potential risk of P release. In this study, chitosan-embedded P-modified biochar (CPBC) was produced for the remediation of Pb(II), Cd(II), and Cr(VI). The specific surface area of CPBC was 5.5 times higher than that of the pristine biochar (BC), and the surface was enriched with functional groups such as -OH and -NH_3_. P-modification facilitated the precipitation of the heavy metals, and chitosan blocked the precipitates inside the biochar. The nature of BC safeguarded the ability to transfer electrons and reduce Cr(VI) to Cr(III), which was further enhanced by the chitosan. Hence, the maximum sorption capacities of CPBC for Pb(II), Cd(II), and Cr(VI) were 29.23%, 129.13%, and 122.12% greater than those of BC. The sequential extraction confirmed that the immobilized Pb(II), Cd(II), and Cr(VI) on CPBC were highly stable, with the sum of acid-soluble and nonbioavailable fractions accounting for 89.14%, 83.73%, and 93.53%, respectively. In addition, chitosan effectively suppressed P release from the P-modified biochar, thereby improving its environmental safety while maintaining excellent heavy metal immobilization performance. The present study demonstrates that CPBC is an effective, environmentally friendly, and universal sorbent to remediate heavy metal pollution in water.

## 1. Introduction

The harm caused to humans by heavy metal (HM) pollution has increased alarmingly as industry continues to grow rapidly [1]. Particularly in certain industrial effluents, the concentration of HMs can be extremely high. For example, the metal ion concentration in plating wastewater can exceed 60 g/L, while the HM concentration in pickling wastewater can surpass 100 g/L [2]. These excessive amounts of heavy metal discharges can potentially endanger environmental safety. HMs are non-biodegradable and persistent in the ecosystem [3,4]. The bioaccumulation and high toxicity of HMs enable their enrichment through food chains, seriously threatening the health and survival of animals, plants, and human beings [5]. Typical HMs, including cadmium (Cd), chromium (Cr), and lead (Pb), can cause human organ damage, skin disease, cancer, mental retardation, growth retardation, etc., after entering the human body [6,7]. The major remediation technologies for HM pollution in water include precipitation, adsorption, ion exchange, and membrane separation [8,9]. Due to its ease of operation, wide availability of materials, and low cost, adsorption is applied universally.

Biochar, a ubiquitous carbon-rich material derived from the pyrolysis of waste biomass, such as wheat straw, woody biomass, waste sludge, and poultry manure under anoxic/hypoxic conditions, has been widely recognized as a green sorbent for immobilizing HMs [10,11]. Its ability to adsorb HMs arises from mechanisms such as surface complexation, precipitation, ion exchange, and electrostatic interaction [12,13,14]. Beyond remediating HM-contaminated sites, applying biochar effectively disposes of agricultural and forestry waste and reduces greenhouse gas emissions, contributing to global carbon fixation [15]. However, pristine biochar suffers from limited sorption capacity and poor recyclability. Moreover, HM immobilization by biochar can be reversed by environmental factors like acid rain and biochar aging, risking HMs’ re-release into the environment [16,17]. Consequently, enhancing biochar’s HM sorption efficiency and enabling its recovery are critical research priorities in this field.

Phosphorus (P)-containing compounds, widely utilized as remediation agents for heavy metal (HM) pollution, can be employed both independently and synergistically with other technologies. P-modified biochar has demonstrated efficacy in forming sparingly soluble precipitates with HM cations [18], thereby mitigating HM migration and associated environmental risks. However, excessive P application poses a risk of water eutrophication. Consequently, it is imperative to control the rapid release of P when integrating P-containing compounds with alternative remediation strategies. Among the available encapsulation strategies, gel-based materials have attracted considerable attention because gels can effectively immobilize functional particles and improve the structural stability and reusability of composite materials [19]. Chitosan, a natural polymer abundant in functional groups [20,21], is one of the most widely used hydrogel-forming biopolymers due to its excellent biocompatibility and facile gelation under mild conditions. Chitosan can form an interconnected three-dimensional network that can encapsulate particulate materials while maintaining the accessibility of reactive functional groups. It is frequently employed as a surface modifier for biochar and other materials. For instance, chitosan-modified kiwifruit branch biochar exhibits a Cd(II) sorption capacity in water that is double that of pristine biochar [22]. Additionally, the adhesive properties of chitosan enable the aggregation and encapsulation of fine biochar particles within its polymeric framework, thereby enhancing biochar recyclability and reusability [23]. Such encapsulation may also provide a barrier that hinders phosphorus release while preserving the accessibility of adsorption sites for heavy metals. However, whether chitosan in the gel state can prevent P release from P-modified biochar and assist biochar in immobilizing HMs remains to be investigated.

It is hypothesized that chitosan could reduce P release and improve the immobilization of HMs on biochar. In this study, chitosan-entrapped potassium phosphate (K_3_PO_4_)-modified straw biochar was used to synthesize composite biochar microspheres for the removal of HMs (Pb, Cd, and Cr) in water. The purposes of this study were (1) to synthesize and characterize a chitosan-embedded P-modified biochar that can effectively immobilize HMs in water; (2) to investigate the mechanisms of HM sorption by the P-modified biochar with chitosan gel; and (3) to analyze the speciation of HMs and evaluate the stability of HMs immobilized by the composite materials.

## 2. Results and Discussion

### 2.1. Selection and Characterization of Materials

**Figure 1:**
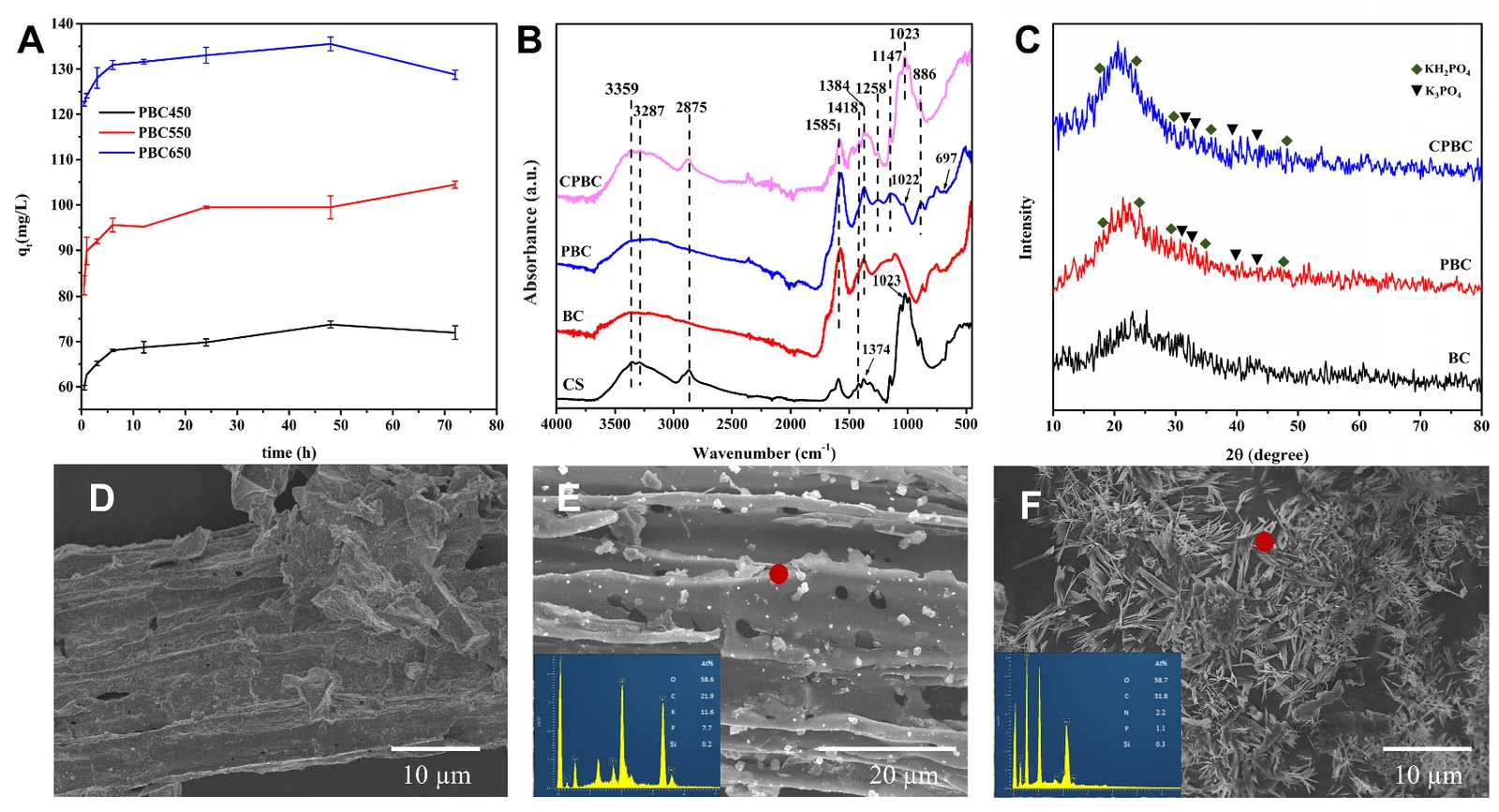
Screening and basic characterization of composite materials. (**A**) P release of P-modified biochar prepared at different temperatures, (**B**) ATR-IR images of CS, BC, PBC, and CPBC, (**C**) XRD images of BC, PBC, and CPBC, (**D**–**F**) SEM images of SEM/EDS images of BC, PBC, and CPBC. The red dots in the SEM images indicate the locations of the EDS point analyses.

The P release from the P-modified biochar at different pyrolysis temperatures is shown in Figure 1A. The P release from PBC at three pyrolysis temperatures reached equilibrium after 6 h. The rapid release of P from PBC at the initial stage may be due to diffusion driven by a concentration gradient [24,25]. This phenomenon shows the potential risk of P being released from the biochar. In addition, the P release from PBC650 was 1.7 times higher than that from PBC450, indicating that the higher pyrolysis temperature reduced the retention of P on the biochar. Therefore, to reduce P release, PBC produced at 450 °C was selected for subsequent experiments in this study.

After modification, the BET surface area of PBC and CPBC increased by 6.95% and 453.97%, respectively (Table 1). Enriched porosity and large specific surface area can improve the sorption capacities of composites for HMs. The zeta potentials of BC, PBC, and CPBC decreased with increasing pH (Table 1, Figure S1). BC (except pH = 4.34) and PBC were both negatively charged while CPBC was positively charged. The -NH_2_ on chitosan is easily protonated to form positively charged -NH_3_^+^ groups at low pH values [26], thus increasing the zeta potential of the CPBC surface.

ATR-IR results showed the stretching vibration peaks of O-H (3359 cm^−1^), C-H (2875 cm^−1^), C-O-C (1023 cm^−1^), and N-H (3287 cm^−1^ and 1418 cm^−1^) on CS [27,28,29] (Figure 1B). After modification with K_3_PO_4_, peaks representing P-O (697 cm^−1^), P-CH_3_ (886 cm^−1^), and *ν*_3_(PO_4_^3-^) (1022 cm^−1^, 1058–1297 cm^−1^) appeared on the PBC [30,31,32,33]. Notably, CPBC had identical types of functional groups compared with CS and PBC, but the intensities of the peaks were lower than those of PBC. XRD confirmed the existence of KH_2_PO_4_ (17.5°, 24.1°, 30.9°, 34.3°, 46.8°) [30,34] and K_3_PO_4_ (30.2°, 31.8°, 33.2°, 38.7°, 44.6°) [35] on both PBC and CPBC (Figure 1C). Therefore, chitosan not only successfully encapsulated the P-modified biochar, but it also maintained the P-containing functional groups and minerals on the biochar surface.

The BC surface exhibited a highly porous structure (Figure 1D). After P modification, some P-containing precipitates (P content > 7.0%) occurred on the smooth biochar surface (Figure 1E). Compared to CS and PBC, CPBC had an irregularly convex surface (Figure 1F), and the surface P content of CPBC (1.1%) was significantly lower than that of PBC. Thus, all the results mentioned above demonstrate the successful production of P-modified biochar microspheres, with chitosan effectively retaining P within PBC and preventing its release into the environment. In addition, the content of elemental C on CPBC remained high (31.8%), a phenomenon that suggests that in addition to elemental C in CS, C in BC may also leach to the surface of CPBC.

### 2.2. Removal of HMs by Composite Materials

#### 2.2.1. Kinetic Studies

**Figure 2:**
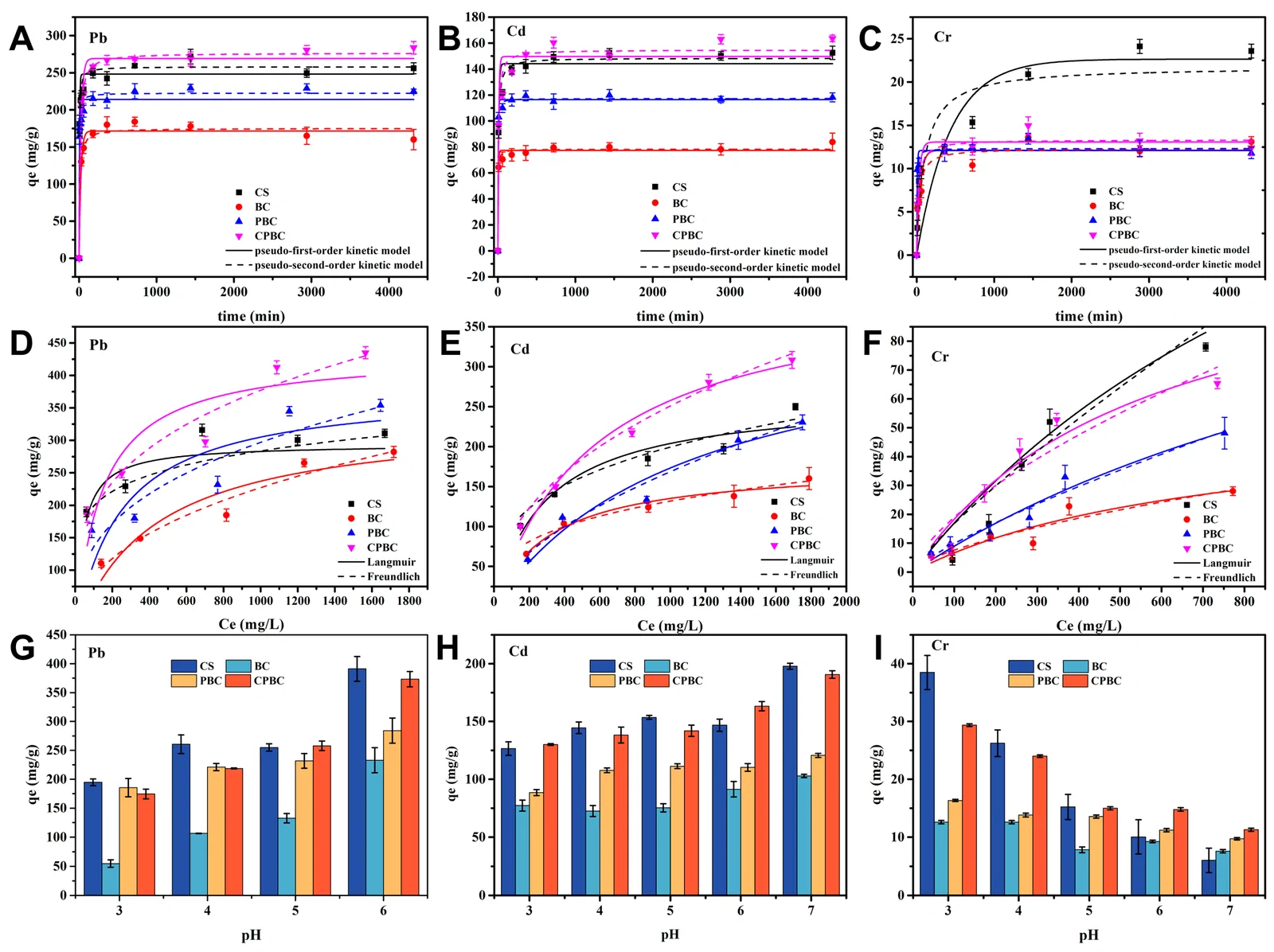
Sorption kinetics (**A**–**C**), isothermal sorption (**D**–**F**), and pH interference (**G**–**I**) results of composites on heavy metal solutions.

The sorption kinetics showed that the sorption of Pb(II), Cd(II), and Cr(VI) onto the sorbents was better described by the PSO model, due to the higher R^2^ values of the PSO models than those of the PFO models. Thus, the sorption of these HMs was dominated by chemical sorption [36]. The sorption of Pb(II) and Cd(II) by all four materials increased rapidly from 0 to 6 h and then reached equilibrium at 12 h (Figure 2A,B). The sorption rates of CPBC and CS were greater than those of BC. The early rapid sorption may be attributed to the large number of reactive sites on the material surfaces [37] and the internal pore diffusion mechanism [38]. Meanwhile, the sorption rates of Cr(VI) with the four treatments were significantly different from those of Pb(II) and Cd(II). After P-modification, the PFO model showed a higher correlation coefficient for PBC than for BC, indicating that the addition of P helped to improve the physisorption performance of biochar on Cr(VI). The adsorption model of CPBC for Cr(VI) was better fitted with improved physical and chemical adsorption characteristics compared to CS and BC (Table 2). Regarding the adsorption of Cr(VI), all the materials were more consistent with the PSO model, which indicates that the adsorption rate of Cr(VI) by these materials was limited by the adsorption sites. PSO rate constants were the largest in the PBC treatment group after adsorption of Cr(VI), which was attributed to the increase in heavy metal adsorption sites by P in PBC. The PSO rate constant of CPBC for Cr(VI) was second only to that of PBC, and it was hypothesized that the CS outside the composite may have facilitated Cr(VI) adsorption. However, the excellent adsorption and retention capacity of CS for Cr(VI) might have limited the diffusion of Cr(VI) to the internal PBC, which led to the reduced adsorption rate of the composite. Although the sorption rates of BC, PBC, and CPBC for Cr(VI) (12 h) were faster than that of CS (24 h), the sorption capacity of CS for Cr(VI) was greater (Figure 2C).

#### 2.2.2. Sorption Isotherms

The Freundlich isotherm model provided a better fit for Pb(II) sorption onto the four sorbents than the Langmuir model (Table 3), indicating that the sorption was multi-layer sorption with heterogeneous sorption sites [39]. Compared with BC and PBC, the maximum sorption capacity of CPBC increased by 29.23% and 14.48%, respectively. However, the sorption behavior of Cd(II) on BC and PBC differed from that of Pb(II). The sorption isotherms showed that the sorption of BC-Cd and PBC-Cd was dominated by monolayer sorption (the higher Langmuir R^2^ values). In addition, the Freundlich model R^2^ for CPBC-Cd was higher than the Langmuir model R^2^ for CPBC-Cd, proving the multi-layer sorption of CPBC-Cd. The Freundlich model R^2^ of CPBC-Cr was smaller than the Langmuir model R^2^, which indicated that the sorption of CPBC-Cr was dominated by monolayer sorption. By integrating the results of sorption kinetics and sorption isotherms, it was implied that the external multilayer, lattice-like structure formed by chitosan contributed to the rapid sorption of HM ions and facilitated their diffusion from the outer layer into the interior of the composite. PBC, acting as an internal sorption carrier, may have provided internal precipitation sites and enhanced HM immobilization within the CPBC. Compared with pre-existing modified biochars, the combination of chitosan encapsulation and P-modification was superior to other modification techniques in enhancing the immobilization of HMs (Table 4).

#### 2.2.3. Effects of pH

The amounts of immobilized Pb(II) and Cd(II) increased with increasing pH for all four materials (Figure 2G,H). When the pH increased from 3 to 6, the sorption of Pb(II) by CPBC and PBC increased by 113.93% and 53.15%, respectively. Meanwhile, the sorption of Cd(II) onto CPBC and CS increased by 17.48% and 56.27% as the pH increased from 3 to 7. At low pH, the surface functional groups were likely to be protonated, causing electrostatic repulsion between the material and cations [50]. As the pH increased, the surface functional groups gradually deprotonated, which facilitated the sorption of metal cations.

Regarding Cr(VI), the Cr(VI) sorption capacity showed an opposite trend compared to Pb(II) and Cd(II) as the pH value increased (Figure 2I). The sorption of Cr(VI) by CS, BC, PBC, and CPBC decreased by 84.36%, 39.71%, 40.38%, and 61.58% as the pH increased from 3 to 7. Some studies have shown that Cr(VI) often exists as oxyanions (HCr_2_O_7_^−^, HCrO_4_^−^, CrO_4_^2−^, Cr_2_O_7_^2−^, etc.), which causes electrostatic attraction between the oxyanions and protonated surfaces [51]. Therefore, the deprotonation of functional groups reduced the positive surface charges on the surface, which reduced the sorption capacity of the sorbents for Cr(VI) [52]. The results confirmed that the zeta potential of CPBC was positive at pH = 3–7, while those of BC and PBC were negative. Hence, CPBC had a greater sorption capacity for Cr(VI) than BC and PBC. When pH > 5, the amount of Cr(VI) adsorbed by CPBC-Cr was higher than that of CS-Cr (Figure 2I). This may be because the electrostatic sorption of CS decreased with increasing pH, whereas the complex surface structure and high specific surface area of CPBC continued to promote the physical sorption of Cr(VI).

### 2.3. The Mechanism of HMs Solidification and Stabilization on Composite Materials

#### 2.3.1. ATR-IR and XRD Analysis

**Figure 3:**
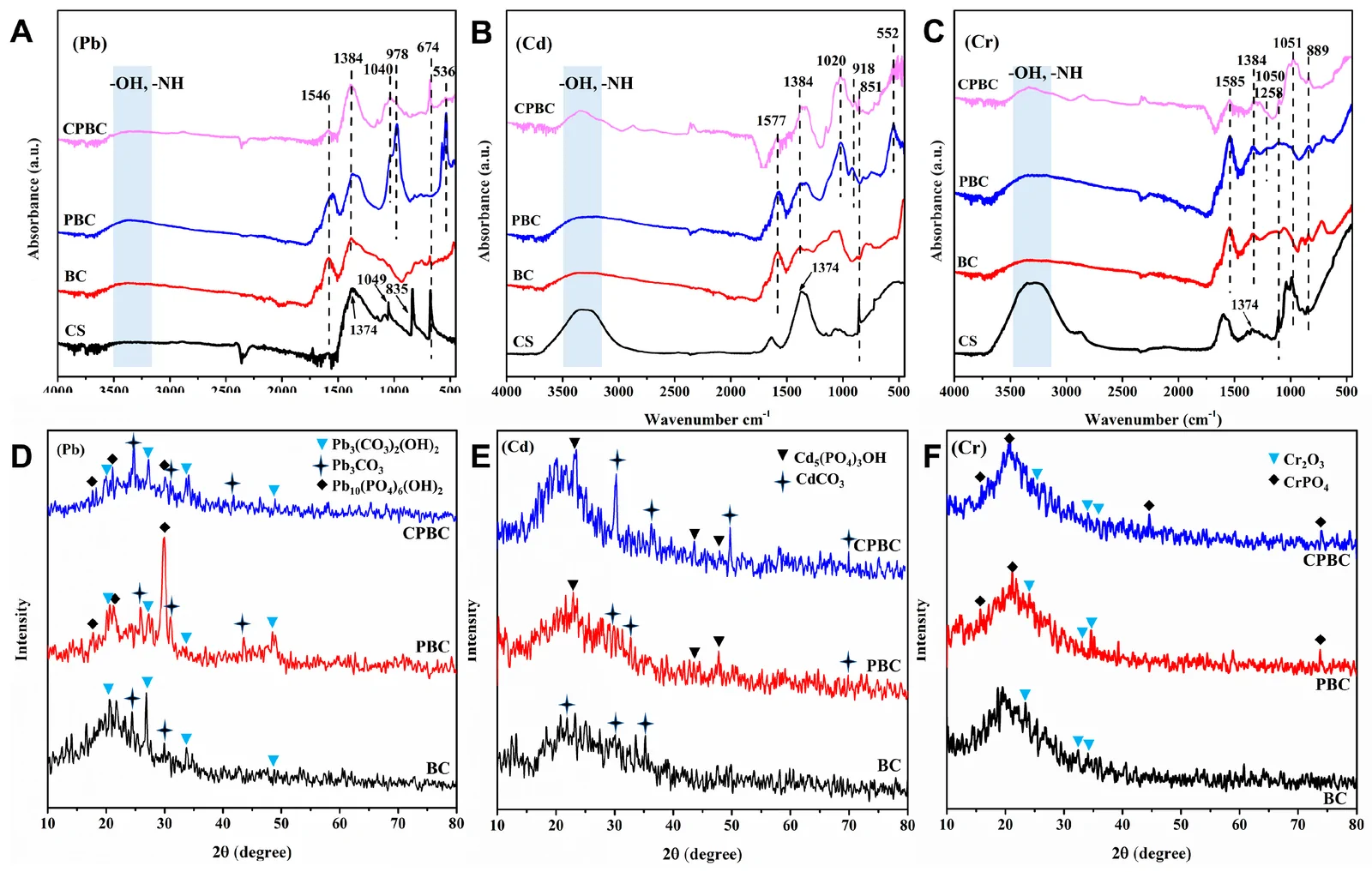
ATR-IR (**A**–**C**) and XRD (**D**–**F**) results of Pb(II), Cd(II) and Cr(VI) sorption on composites.

The ATR-IR results are shown in Figure 3A–C. After adsorption of Pb(II) and Cd(II), the carboxyl group characteristic peaks (1585–1595 cm^−1^) on BC, PBC, and CPBC underwent a shift towards lower wavenumbers and decreased in intensity [30]. Disappearance of the amino groups (1418 cm^−1^) occurred on CPBC and CS, and a decrease in the intensity of the hydroxyl groups (~3359 cm^−1^) occurred on all materials. These phenomena indicate that the carboxyl [53], amino, and hydroxyl groups [22] on biochar and chitosan can assist in chelating and precipitating HMs. The characteristic peaks of PO_4_^3−^ on PBC (536, 978, 1040, 1058–1297 cm^−1^) and CPBC (552, 1022 cm^−1^) after the sorption of Pb(II) and Cd(II) were stronger than those on BC [30,54,55], which may be attributed to the co-precipitation of phosphate with Pb(II) and Cd(II) on P-modified biochar.

The types of functional groups involved in for Cr(VI) sorption were similar to those of Pb(II) and Cd(II) sorption. The intensities of carboxyl groups (1585 cm^−1^) of BC, PBC, and CPBC decreased after adsorbing Cr(VI), indicating that the carboxyl groups underwent deprotonation and formed chemical bonds with Cr(VI) [56]. The characteristic hydroxyl band on CPBC-Cr and CS-Cr formed a broad peak (3200–3400 cm^−1^) at the location of the characteristic amino band (3359 cm^−1^), which may be attributed to the hydroxyl group on chitosan broadening the band through coordination with Cr(VI) [57]. The shift of the amino band also suggests the complexation of Cr(VI) [58]. It is noteworthy that the peak representing Cr (1051 cm^−1^) on CS was significantly stronger than that on PBC, indicating a stronger affinity of CS for Cr(VI) than that of PBC, which is consistent with the sorption results.

The XRD results confirmed the presence of new minerals after sorbing HMs (Figure 3D–F). On BC, carbonate minerals such as Pb_3_(CO_3_)_2_(OH)_2_, PbCO_3_, and CdCO_3_ were observed [17,59,60]. However, phosphate-related peaks such as Pb_10_(PO_4_)_6_(OH)_2_ and Cd_10_(PO_4_)_6_(OH)_2_ dominated on PBC and CPBC [46,61]. Additionally, the intensities of the peaks of Pb phosphates on PBC and CPBC were significantly stronger than those of Cd phosphates, which was probably due to the lower solubility product constant (K_sp_) of Pb phosphate (K_sp_ = 8.0 × 10^−43^) compared to Cd phosphate (K_sp_ = 10^−33^–10^−30^); thus, it precipitated more easily [62]. This can also explain the higher sorption capacity of PBC and CPBC for Pb(II) than for Cd(II) (Figure 2G,H). Cr(III)-containing minerals also occurred on BC, PBC, and CPBC (Figure 3F), indicating the reduction of Cr(VI) on BC and chitosan surfaces, followed by the precipitation and complexation of Cr(III) [63]. The reduction of Cr(VI) to Cr(III) is likely to have been coupled with oxidation of electron-donating functional groups on the sorbent, e.g., phenolic−OH and quinone-type moieties on biochar [64]. At relatively low pyrolysis temperatures (<600 °C), electron-donating moieties are considered the primary electron donors, whereas aromatic structures become increasingly important at higher pyrolysis temperatures [65]. In the CPBC composite, chitosan is more likely to facilitate Cr(VI) reduction by enriching Cr(VI) at the sorbent surface through electrostatic attraction and complexation, thereby promoting electron transfer from the biochar matrix, rather than acting as the primary electron donor itself. Compared with PBC and CPBC, only a few heavy metal carbonate phases were observed in the XRD pattern of BC, which may imply that the adsorption of Cr by BC was mostly dependent on pore filling and surface complexation (Figure 3C and Table 2).

#### 2.3.2. Morphology and HMs Distribution

**Figure 4:**
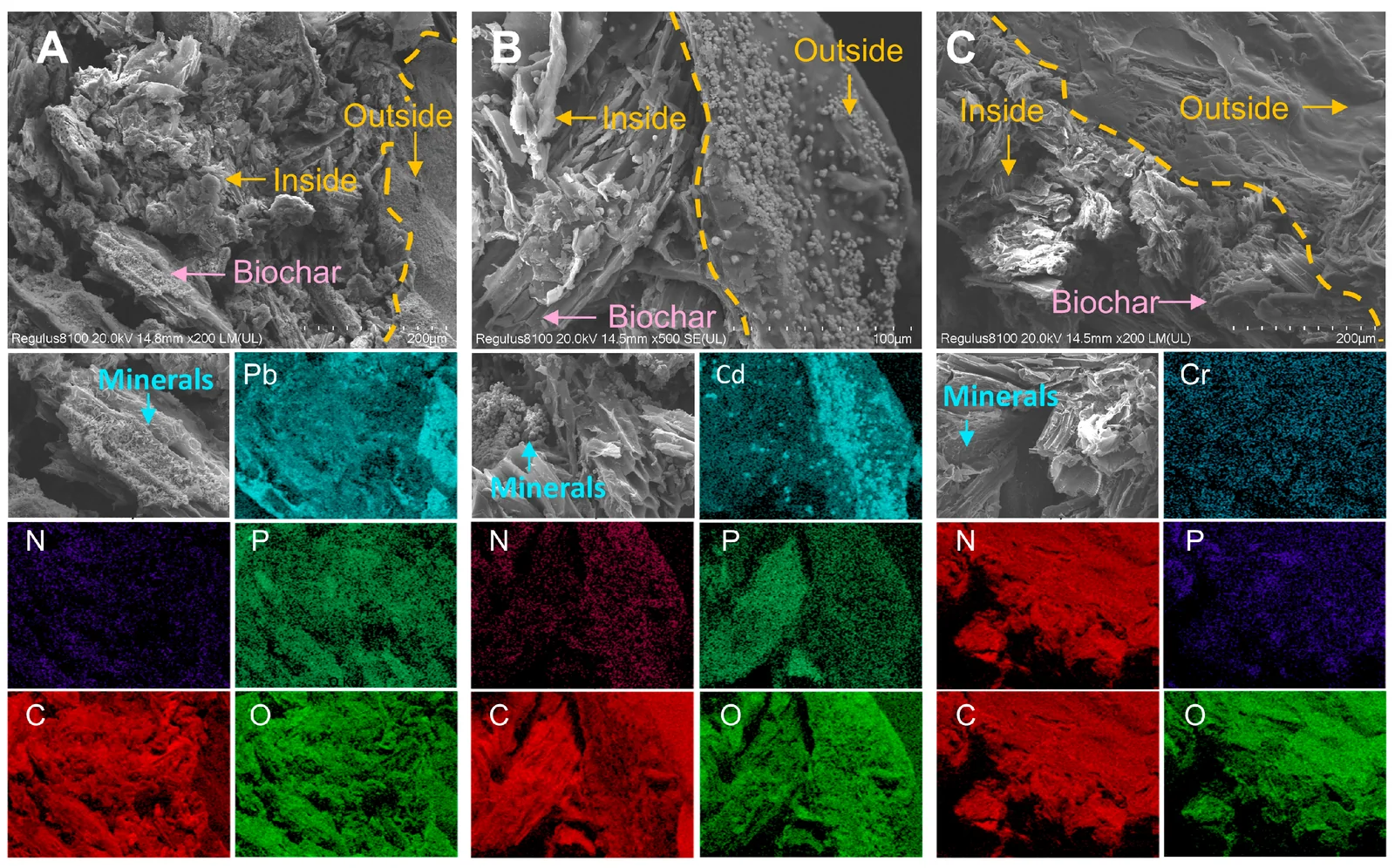
SEM/EDX results of CPBC after sorption of Pb(II) (**A**), Cd(II) (**B**) and Cr(VI) (**C**).

The SEM results showed porous and rod-shaped biochar particles in CPBC (Figure 4). The EDX results showed that the signals of HMs were stronger on the surface than inside the CPBC. However, HM elemental signals were detected on the biochar particles inside the CPBC, while HMs signals in the pores were not significant. This phenomenon suggests that HMs can migrate through the pores of the outer chitosan layer and be immobilized by biochar instead of chitosan. Uniform micro-particles mainly occurred on the surface of CPBC after immobilizing Pb(II), while layered and needle-like minerals were mainly observed on the internal biochar (Figure 4A). The overlap of P, Pb, and O signals indicated the presence of pyromorphite, one of the most stable Pb-containing minerals. Nevertheless, the surface of CPBC-Pb showed high contents of Pb, O, and C (54.4%, 21.0%, and 24.6%, respectively), indicating that the minerals on the surface were likely to have been carbonates (Figure S2A).

After adsorption of Cd(II), granular minerals were observed on the surface and inside of the CPBC (Figure 4B). As Cd highly overlapped with C, O, and P under EDX, the Cd(II) carbonates and phosphates on the CPBC surface were verified. Similar to after Pb(II) sorption, the surface of the CPBC was covered with abundant mineral precipitates, with a higher carbon content (16.2%) than P content (0.3%). Therefore, it can be inferred that the surface minerals were also carbonates (Figure S2B). Notably, no mineral particles were observed on the CPBC after sorbing Cr(VI) (Figure 4C). However, the overlap of signals for P and Cr(VI) within CPBC indicated that the presence of P might have facilitated the sorption and precipitation of Cr(VI), consistent with ATR-IR and XRD results. In addition, SEM results indicated that the nitrogen (N) signals did not correspond to HM elements (Figure S2). Therefore, the N-containing functional groups in chitosan were not critical in the precipitation of HMs.

### 2.4. Influence of HM Sorption on Electrochemical Response

The CV curves of BC, PBC, and CPBC before sorption all exhibited characteristic oxidation and reduction peaks (Figure S3A–C), which may have been caused by functional groups with oxidizing or reducing nature [66]. The electrochemically active surface area A of the Randles Sevcik model (A_o_: oxidized electrochemically active surface, A_R_: reduced electrochemically active surface) of a material reflects the electron transfer ability of the material [67]. Studies have shown that the N on CS has the ability to trap electrons [68], and the abundant O-containing functional groups on biochar can accept electrons and transfer them to other substances [26]. The trends of both oxidized and reduced electrochemically active surface areas were in the order of CPBC > BC > PBC (Figure S3A–C), indicating that the chitosan embedding contributed greatly to the electrochemically active surface area of BC.

**Figure 5:**
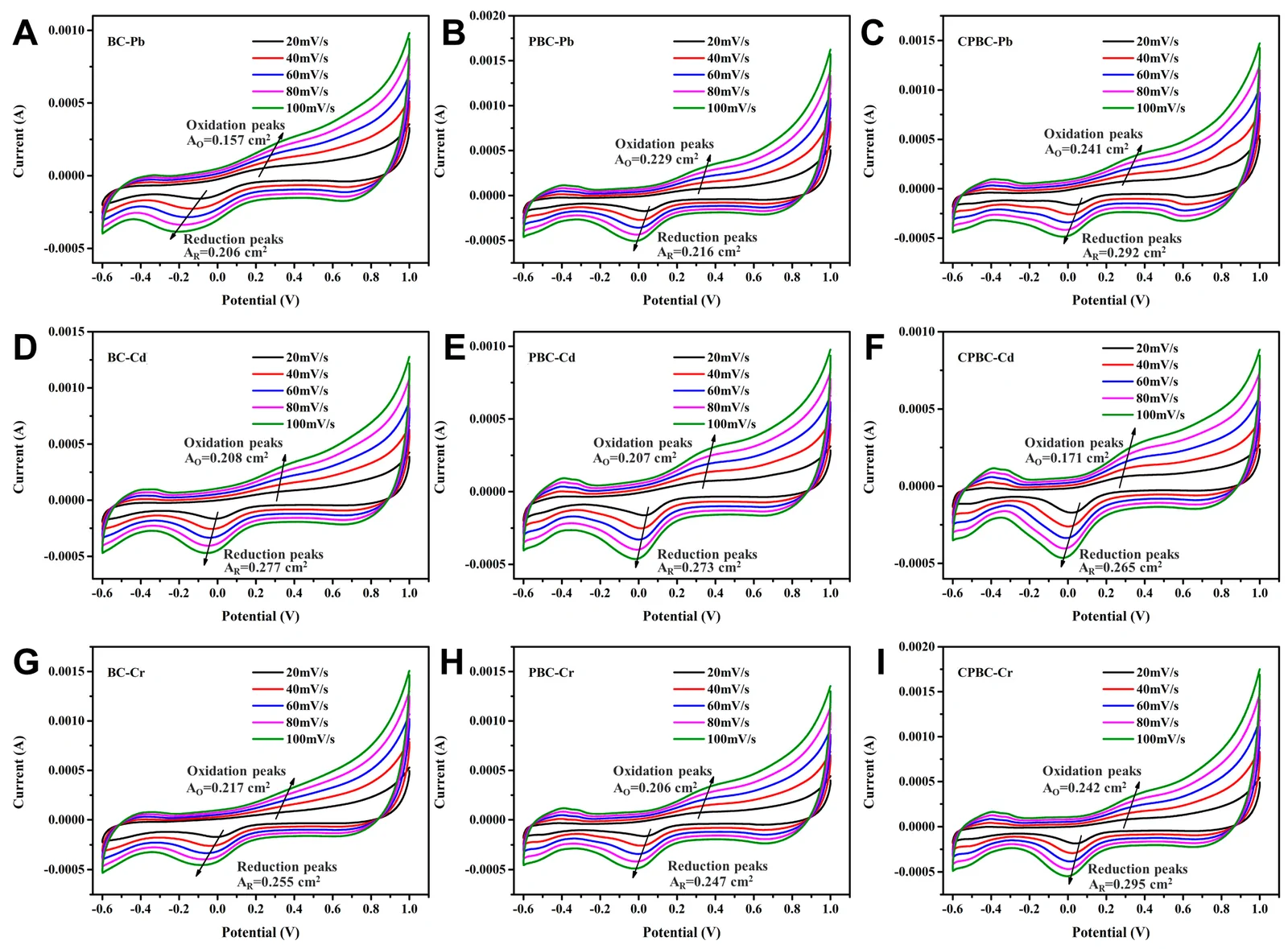
The CV curves for sorption of Pb(II) (**A**–**C**), Cd(II) (**D**–**F**), and Cr(VI) (**G**–**I**) by BC, PBC, and CPBC.

The electrochemical-active surface area A increased for all three materials after Pb(II) sorption. The A_O_ and A_R_ values were 5.24–53.50% higher in CPBC-Pb than those in BC-Pb and PBC-Pb, respectively (Figure 5A–C). This indicates that the electron transfer capacity of CPBC was greater than that of BC and PBC after Pb(II) sorption. It has been shown that the formation of minerals on biochar leads to a reduction in electron transfer capacity [69]. The PBC inside the CPBC can attract and immobilize Pb(II) by forming stable Pb-containing phosphates, and the minerals did not detach from the biochar and blocked the pores on the CPBC (Figure 4), which maintained the electron transfer on the CPBC.

After the sorption of Cd(II), the A_O_ and A_R_ values of CPBC-Cd were smaller than those of PBC-Cd and BC-Cd (Figure 5D–F). Thus, the electron transfer capacity of CPBC-Cd was lower than that of PBC-Cd and BC-Cd. The combination of SEM and XRD results reveals that some of the granular Cd-carbonate may have been detached from the biochar, causing pore blockage. Meanwhile, functional groups such as hydroxyl groups on the CPBC surface may have further reduced the active sites through surface precipitation and ion exchange [67], thus reducing the electrochemical electron transfer capacity.

**Figure 6:**
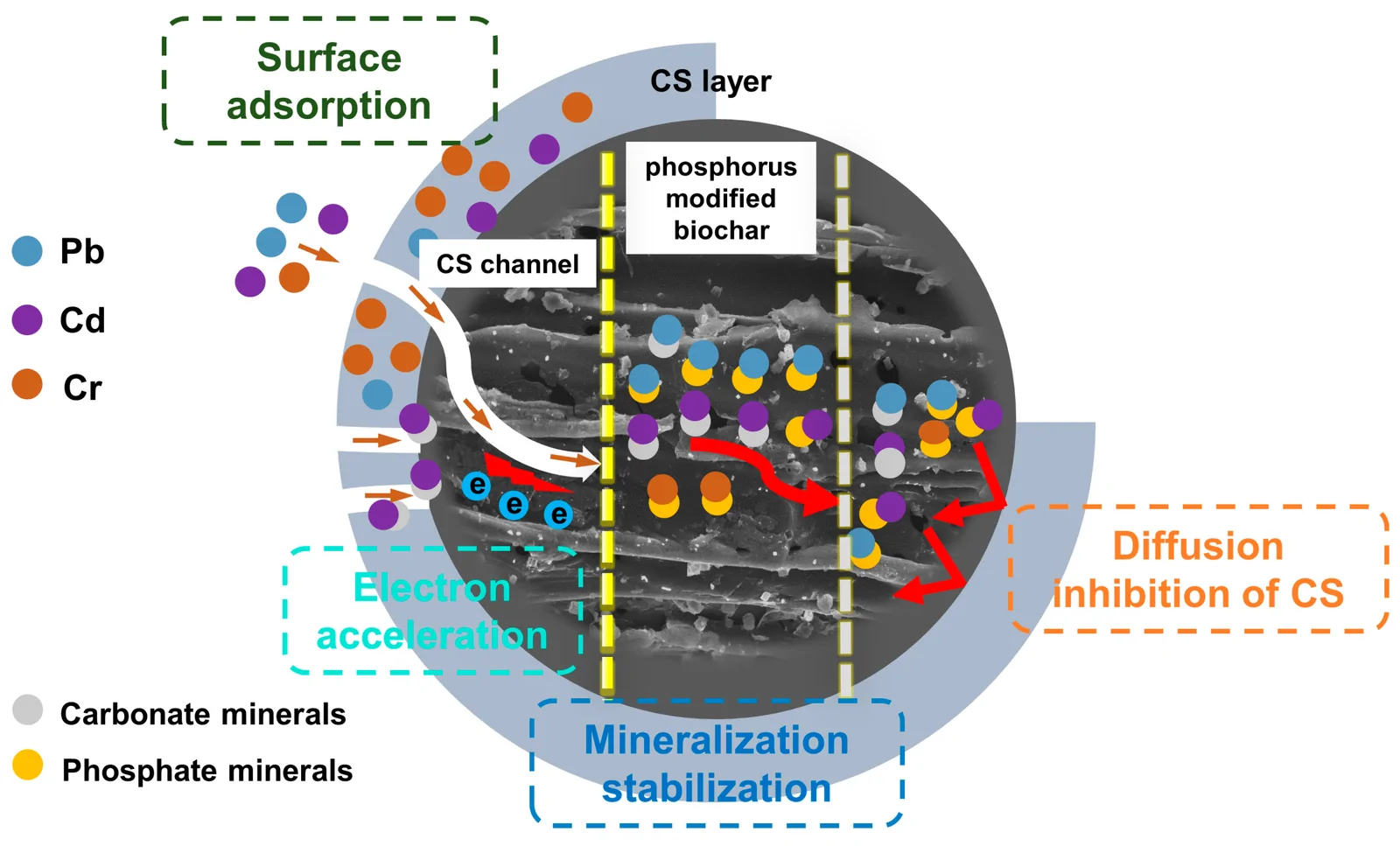
Stabilization mechanism of mineral solidification of CPBC.

The A_O_ and A_R_ values were higher in CPBC-Cr than those in BC-Cr and PBC-Cr (Figure 5), which also verified that the contribution of CS to Cr(VI) sorption was greater than that of P modification (Figure 2C,F). The electron transfer ability of biochar can reduce Cr(VI) by donating electrons to Cr(VI) [70]. Meanwhile, the positive charge provided by CS can effectively reduce the electrostatic repulsion between Cr(VI) and CPBC, thus promoting the migration of Cr(VI) from the aqueous phase to CPBC. SEM results showed that no obvious mineral crystals were observed on the surface and pores of CPBC-Cr (Figure 4C). Therefore, a smooth CS channel connected the exterior to the interior of the material, thus enhancing the electron transfer process and facilitating the migration of Cr(VI) to the interior of the CPBC. In conclusion, the HM transport pathways formed by the outer CS and the sorption platform provided by the inner PBC combined to facilitate the sorption of Pb(II), Cd(II), and Cr(VI) by the CPBC (Figure 6). HMs were rapidly sorbed by the CS in the outer layer of the CPBC and formed complexes on the surface which were then transported to the interior of the CPBC through the pores of the CS. The HMs were immobilized in the form of carbonates and phosphates inside the CPBC. The CS layer on the surface of the CPBC encapsulated the HMs, preventing their dissolution into the environment and where they would cause secondary pollution.

### 2.5. Risk Analysis of Composite Materials After HMs Sorption

**Figure 7:**
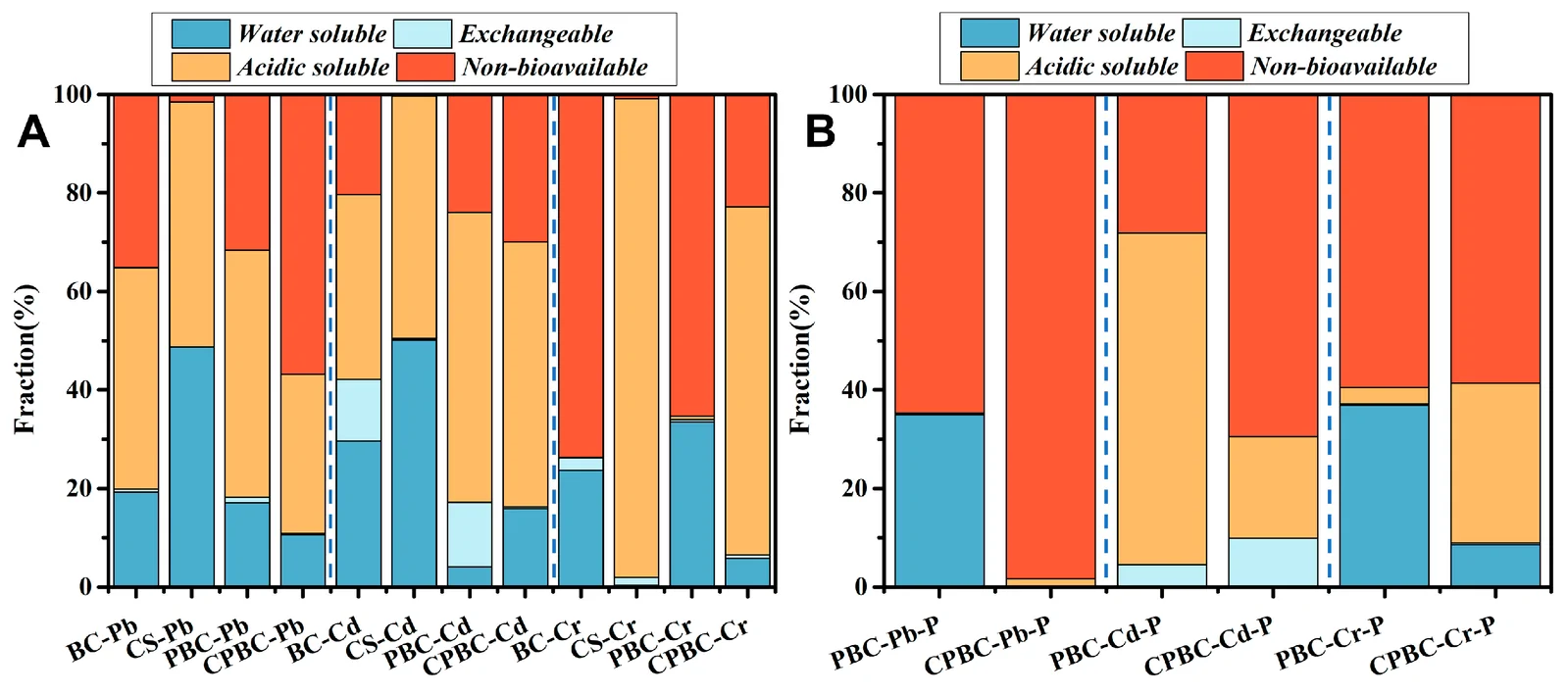
Speciation of Pb(II), Cd(II), and Cr(VI) (**A**) and P (**B**) on biochars.

The results of the four-step sequential extraction of BC, CS, PBC, and CPBC after HMs sorption are shown in Figure 7A. The water-soluble fraction of all biochar materials (BC, PBC, and CPBC) after Pb(II) sorption was relatively low (10.57–19.31%), while that of the CS reached 48.71%. The high proportion of water-soluble fraction indicates that the Pb(II) immobilized by CS was unstable, posing a high environmental risk. Although the non-bioavailable fractions of BC-Pb and PBC-Pb were similar, the non-bioavailable fraction Pb(II) was 26.39% higher in PBC than in BC (Table S2). Pyromorphite is likely to have contributed to the non-bioavailable Pb(II) on PBC, owing to its low solubility [71]. Additionally, the non-bioavailable fraction of Pb(II) on CPBC (56.83%) was much greater than that on CS (1.55%) and BC (35.18%). Similarly, the acid-soluble (58.80%) and non-bioavailable fractions (24.00%) of Cd(II) on PBC were also higher than those on BC. Compared to PBC, CPBC showed a slight increase in the water-soluble fraction of Cd(II) after sorption, which was likely to be attributable to the physical sorption of Cd(II) by chitosan [72]. However, the content of acid-soluble and non-bioavailable fractions of Cd(II) in CPBC were further increased, being 1.27 times and 1.73 times higher than PBC, respectively (Table S2).

The total percentage of acid-soluble and non-bioavailable fractions of Cr(VI) in PBC-Cr was 7.76% lower than that of BC-Cr. P modification could not improve the immobilization of Cr(VI) by biochar. After sorption of Cr(VI) on CS, the water-soluble fraction was 0.37% and the acid-soluble fraction was 97.19%. CPBC had a lower water-soluble fraction of Cr(VI) than BC and PBC (Table S2), indicating that the addition of chitosan decreased the mobility of Cr. The acid-soluble Cr(VI) (70.71%) on CPBC was significantly higher than that on BC and PBC, but the non-bioavailable Cr(VI) (22.81%) was low compared to BC and PBC. Combined with the CS-Cr extraction results, the increase in the acid-soluble fraction in CPBC-Cr was mainly attributed to the presence of CS. Consequently, CPBC exhibited a better ability to immobilize Pb(II), Cd(II), and Cr(VI) compared to BC and PBC because it had a higher sum of acid-soluble and non-bioavailable Pb(II) (89.14%), Cd(II) (83.73%), and Cr(VI) (93.53%). The efficiency of CPBC in immobilizing HMs was in the order Pb(II) > Cd(II) > Cr(VI) (Table S2).

Furthermore, four-step sequential extraction results for P in PBC and CPBC also indicated that most P was non-bioavailable (Figure 7B). The non-bioavailable fractions of P on CPBC after Pb(II) and Cd(II) sorption were 33.63% and 41.35% higher than those on PBC (Figure 7B). Although the non-bioavailable P was nearly identical on PBC and CPBC after Cr(VI) sorption (Figure 7B), the acid-soluble P on CPBC was 29.20% higher than that on PBC. Correspondingly, CPBC had less water-soluble P. The presence of P not only formed stable phosphates with Pb(II) and Cd(II) but also promoted the formation of more stable acid-soluble species with Cr(VI) (e.g., CrPO_4_), greatly enhancing the sorption stability of the composite material for HMs. Moreover, compared with chitosan modification, improving chemical complexation/precipitation was generally more effective in enhancing the immobilization of HMs.

Previous studies have shown that chitosan-based hydrogels can effectively remove heavy metals mainly through amino and hydroxyl functional groups, electrostatic attraction, and complexation [20]. In contrast, the CPBC developed in the present study integrated these adsorption mechanisms with P-induced mineral and the redox activity of biochar, resulting in higher stability of immobilized HMs, as evidenced by the increased acid-soluble and non-bioavailable fractions obtained from sequential extraction. Moreover, unlike conventional chitosan hydrogels that mainly function as adsorption matrices, the chitosan gel in CPBC also serves as an encapsulation network that suppresses phosphorus release while facilitating the synergistic immobilization of both cationic and anionic heavy metals.

## 3. Conclusions

In this study, P-modified biochar microspheres were synthesized by chitosan encapsulation. Sorption experiments and characterization verified their efficiencies in immobilizing Pb(II), Cd(II), and Cr(VI) in water. The sorption capacity of CPBC for Pb(II), Cd(II), and Cr(VI) increased by 29.23%, 129.13%, and 122.12% compared with pristine biochar, and was also greater than that of P-modified biochar; these results are ascribed to the large specific surface area and enriched surface functional groups of CPBC. The addition of P ensured the precipitation of Pb(II) and Cd(II). The formation of sparingly soluble phosphates contributed greatly to the stabilized fractions of Pb(II) and Cd(II) on CPBC (~90% acid-soluble and non-bioavailable fractions). The chitosan gel mediated the sorption of HMs, which not only prevented P release but also enhanced sorption by electrostatic attraction and electron transfers. Although Cr(VI) lacks stable minerals, the high proportion of stable Cr fractions on CPBC indicated that chitosan gel can concentrate Cr on the CPBC, which facilitated the transport of Cr to the inner CPBC under a high concentration gradient. Furthermore, the presence of Cr(III) minerals confirmed the ability of CPBC to reduce Cr(VI), inhibiting the mobility and toxicity of Cr. Overall, CPBC represents a promising and recyclable sorbent for remediating both cationic and anionic heavy metal species. This study demonstrated that chitosan encapsulation and P modification act synergistically to enhance both the adsorption performance and immobilization stability of HMs. The composite provides an effective strategy for simultaneously controlling phosphorus release and improving the remediation of both cationic and anionic heavy metal contaminants. Future studies should further investigate the long-term stability and regeneration performance of the composite under practical environmental conditions.

## 4. Materials and Methods

### 4.1. Preparation of the Materials

Corn straw was used as the raw material for biochar. Before the experiment, it was ground until the particle size was below 0.27 mm. This study refined the preparation conditions of P-modified biochar according to previous studies [42]. The screened corn straw and K_3_PO_4_ (1:0.5 *w*/*w*) were soaked and stirred at 50 °C for 3 h. Then, the mixed solution was subjected to solid–liquid separation and drying (60 °C, 5 h). The dried solid was pyrolyzed in a muffle furnace (450/550/650 °C, heating rate 5 °C/min, maintaining the maximum temperatures for 2 h) to obtain P-modified biochar (PBC450, PBC550, and PBC650).

Then, 1 g PBC was added to 60 mL of a 2% (*v*/*v*) acetic acid solution containing 1 g of chitosan and stirred at 25 °C for 30 min to obtain mixed solution A. The P-modified biochar microspheres (designated CPBC450, CPBC550, CPBC650) were produced by dropping the mixed solution A into the NaOH (1.2%, *w*/*w*) solution with a syringe and soaking for 12 h. The obtained CPBC microspheres were washed three times with deionized water (removing excess P on the surface) and dried at 60 °C for standby. In addition, chitosan microspheres (CS) were prepared with the same method. Pristine corn straw biochar (BC), prepared at the corresponding pyrolysis temperatures without P modification, was used as the control.

Analytical grade chitosan (Sinopharm Chemical Reagent Co., Ltd., Shanghai, China; viscosity-average molecular weight: 150–200 kDa; degree of deacetylation: 85–90%) was used in this study. Analytical grade nitric acid (HNO_3_), lead nitrate [Pb(NO_3_)_2_], magnesium chloride (MgCl_2_), sodium acetate (CH_3_COONa), potassium dichromate (K_2_Cr_2_O_7_), sodium hydroxide (NaOH), and cadmium nitrate tetrahydrate [Cd(NO_3_)_2_·4H_2_O] were purchased from Sinopharm Chemical Reagent Co., Ltd. (Shanghai, China). Analytical grade glacial acetic acid (CH_3_COOH) was purchased from Meryer (Shanghai, China). Unless otherwise specified, all reagents were of analytical grade and used without further purification. Deionized water was used throughout the experiments.

### 4.2. Experimental Methods

In this study, BC-Pb/Cd/Cr, CS-Pb/Cd/Cr, PBC-Pb/Cd/Cr, and CPBC-Pb/Cd/Cr denote the treatments that used BC, CS, PBC, and CPBC to immobilize Pb(II), Cd(II) and Cr(VI), respectively.

#### 4.2.1. Selection of Materials

For this research, 0.2 g of PBC450, PBC550, and PBC650 were added to 250 mL conical flasks containing 100 mL of deionized water and shaken at 25 °C (200 r/min). The supernatant was withdrawn using a syringe every 0.5, 1, 3, 6, 12, 24, 48, and 72 h, then filtered through a 0.22 µm membrane filter. The total soluble phosphorus content in solution was determined using molybdenum blue spectrophotometry. Lower soluble P in the solution indicated more stable P on the biochar. In this study, the composite preparation conditions with the lowest total soluble phosphorus content were selected for subsequent experiments.

#### 4.2.2. Sorption Experiment

In this study, the sorption kinetics, sorption isotherms, and pH edges of all types of CS, BC, PBC, and CPBC were investigated, respectively. The initial concentrations of Pb(II), Cd(II), and Cr(VI) were selected based on preliminary experiments and previous studies to provide sufficient adsorption rates for adsorption evaluation. All adsorption experiments were conducted in 0.01 M NaNO_3_. In these experiments, 0.1 g of material was added to a triangular flask containing 100 mL of HM solution for sorption kinetic experiments (Pb(II) = 1000 mg/L, Cd(II) = 500 mg/L and Cr(VI) = 200 mg/L, pH = 5). The triangular flask was shaken for 72 h (200 r/min, 25 °C). The suspension was sampled and filtered after 0.5, 1, 3, 6, 12, 24, 48, and 72 h, respectively. In addition, 0.1 g of material was added to a triangular flask containing 100 mL of HM solutions with different concentrations for the sorption isotherm experiments (Cd(II)/Pb(II) = 250, 500, 1000, 1500 and 2000 mg/L, Cr(VI) = 50, 100, 200, 400 and 800 mg/L, pH = 5). The triangular flask was shaken at 200 r/min at 25 °C for 24 h. Then, the suspensions were sampled and filtered. The pseudo-first-order (PFO) and pseudo-second-order (PSO) models were used to analyze the sorption kinetics [73]. The Langmuir and Freundlich isotherms were fitted [46]. The corresponding equations of adsorption kinetic models and isotherms, the equilibrium adsorption capacity (Q_e_e), and the removal rate (η) of each adsorbent for Pb(II)/Cd(II)/Cr(VI) are listed in Table S1.

Another 0.1 g of material was added to a triangular flask containing 100 mL of HM solution with different pH values (pH = 2, 3, 4, 5, 6, 7, Pb(II) = 1000 mg/L, Cd(II) = 500 mg/L, and Cr(VI) = 200 mg/L). To avoid the precipitation of Pb(OH)_2_, the pH range of the Pb(II) solution was set to 2–6 according to preliminary tests. The initial pH value of the solution was adjusted with HNO_3_ (0.1 M) and NaOH (0.1 M) solutions. The suspensions were shaken at 200 r/min for 24 h. The suspension was filtered after sorption through a 0.22 µm filter membrane, and then, the concentrations of HMs were determined. Three replicates were performed for all experiments. The chitosan microspheres and CPBC microspheres remained structurally intact throughout the pH range (pH 2–7), and no noticeable disintegration or dissolution was observed during the adsorption experiments.

### 4.3. Electrochemical Analysis

The electrochemical properties of the samples were analyzed with an electrochemical workstation (CHI760E, CH Instrument Company, China, ShangHai). The conditions were set to Init E (V) = 1, High E (V) = 1, Low E (V) = −0.6, and Sensitivity (A/V) = 0.001. The reference electrode was a saturated calomel electrode (SCE, saturated KCl internal solution), the auxiliary electrode was a platinum wire, and the working electrode was a homemade clip electrode. The working electrode consisted of a high-purity nickel sheet (effective geometric surface area: 1.0 cm^2^) used as the conductive substrate. The dried sorbents were ground and attached to nickel sheets. Then, they were fixed by the clamp electrode and then measured in an electrochemical cell containing phosphate buffer (herein, 0.2 M phosphate buffer, pH = 6.5–7.6, 27 °C). The cyclic voltammetry curves (CV) of the samples were measured at different scanning rates (Scan Rate (V/s) = 0.02, 0.04, 0.06, 0.08, 0.1). The electrochemically active surface area of the Electrode (A) was calculated using the Randles-Sevcik model [67], as follows:

(1) $$I_{p}=(2.69\times 10^{5})n^{1.5}AD^{0.5}Cv^{0.5}$$

where *I_p_* is the peak current, *A* is the electrochemical active surface area, *C* is the concentration of electrolyte solution, *v* is the scan rate, *D* is the diffusion coefficient, and *n* is the charge transfer number (*n* = 1). The electrochemical active surface area of reactants can be represented by the slope of the curve (*I_P_*/*v*^1/2^).

### 4.4. Sequential Extraction of HMs

The four-step sequential extraction method was used to determine the stability of HMs and P in the sorbents (water-soluble, exchangeable, acid-soluble, and non-bioavailable fractions). The four-step extraction method involved sequential extraction of the samples using four extraction solutions (deionized water, 0.5 M MgCl_2_, 1 M NaOAc-HOAc, aqua regia) [74].

### 4.5. Characterizations

The element contents (C, S, N) of the material were quantified using an element analyzer (Vario EL III, Elementar Analysensysteme GmbH, Langenselbold, Germany). A pH meter (SG98 InLab, Mettler Toledo International, Greifensee, Switzerland) was used to determine the pH values. The concentrations of Pb(II), Cd(II), and Cr(VI) in the solution were determined by inductively coupled plasma optical emission spectrometry (ICP-OES, Agilent 710, Agilent Technology Co. Inc., Santa Clara, CA, USA). An attenuated total reflectance infrared spectroscope (ATR-IR, Nicolet iS5 Fourier transform infrared spectrometer, Thermo Fisher Scientific Inc., Madison, WI, USA.) equipped with a diamond ATR crystal was used to identify the functional groups of the material (wavenumber range 500–4000 cm^−1^). A specific surface area and porosity analyzer (Micro Active for ASAP 2460 2.01, Micromeritics Instrument Corporation, Norcross, GA, USA) was used to determine the Brunauer–Emmett–Teller (BET) surface areas of the materials under N_2_ adsorption at 77 K. The surface structure of the material was measured using a scanning electron microscope equipped with energy-dispersive spectroscopy (SEM-EDS, Zeiss sigma300, Oberkochen, Germany; 5 kV), and information about the elemental content of the materials was obtained via EDS/EDX with an InLens probe (15 kV). The precipitates were identified using an X-ray powder diffractometer (XRD, D8 Advance, Bruker AXS GMBH, Karlsruhe, Germany, 40 kV, and 40 mA, Cu Kα radiation with λ of 1.5406 Å, scanning range: 10–80°, scanning rate: 0.1 s/step).

### 4.6. Statistical Analysis

XRD and FTIR data analyses were carried out using JADE 6.0 and Thermo Scientific OMNIC software 9.2, respectively. Origin 8.0 was used for fitting curves and plotting.

## Figures and Tables

**Table 1:** The properties of materials.

Biochar	BET Surface Area (m^2^/g)	Elemental Composition (%, Mole Based)			C/N Molar Ratio	Zeta Potential (mV) pH = 3~7
		C	S	N		
BC	3.2669 ± 0.0261	49.03 ± 0.83	0.060 ± 0.00168	0.96 ± 0.0202	50.92	7.28~−43.50
PBC	3.4938 ± 0.0384	42.39 ± 0.97	0.110 ± 0.00154	0.81 ± 0.00324	52.02	−5.08~−30.70
CPBC	18.0975 ± 0.4705	45.11 ± 0.27	0.130 ± 0.00117	3.96 ± 0.0752	11.39	46.20~20.10

**Table 2:** Fitted parameters of Pb(II), Cd(II), and Cr(VI) sorption kinetics.

Metal	Samples	Pseudo-First Order			Pseudo-Second Order		
		k_1_ (min^−1^)	Q_e_ (mg/g)	R^2^	k_2_ (g/mg min)	Q_e_ (mg/g)	R^2^
Pb	CS	0.172 ± 0.039	248.18 ± 7.67	0.869	0.001 ± 0.0002	258.05 ± 5.32	0.935
	BC	0.043 ± 0.006	171.55 ± 3.71	0.974	0.001 ± 0.0001	175.02 ± 4.02	0.976
	PBC	0.237 ± 0.055	214.06 ± 5.94	0.895	0.002 ± 0.0004	222.59 ± 4.18	0.953
	CPBC	0.040 ± 0.005	269.18 ± 5.32	0.978	0.001 ± 0.0001	276.62 ± 2.67	0.996
Cd	CS	0.098 ± 0.019	144.14 ± 3.80	0.959	0.001 ± 0.0001	148.44 ± 2.28	0.988
	BC	0.178± 0.033	77.34 ± 1.55	0.975	0.005 ± 0.0015	78.27 ± 1.35	0.983
	PBC	0.216 ± 0.024	116.46 ± 1.17	0.996	0.005 ± 0.0012	117.25 ± 0.96	0.996
	CPBC	0.101 ± 0.027	149.60 ± 5.50	0.924	0.001 ± 0.0002	154.68 ± 4.21	0.964
Cr	CS	0.002 ± 0.001	22.64 ± 2.52	0.784	0.001 ± 0.0002	21.79 ± 1.91	0.869
	BC	0.022 ± 0.007	12.12 ± 0.85	0.864	0.003 ± 0.0011	12.34 ± 0.71	0.916
	PBC	0.162 ± 0.004	12.08 ± 0.38	0.955	0.027 ± 0.0088	12.32 ± 0.30	0.976
	CPBC	0.032 ± 0.008	13.04 ± 0.63	0.923	0.004 ± 0.0012	13.34 ± 0.55	0.948

**Table 3:** Fitted parameters of Langmuir and Freundlich isotherms for different materials.

Metal		Freundlich Model			Langmuir Model		
		K_F_	n	R^2^	K_L_	Q_max_	R^2^
Pb	CS	102.17 ± 25.22	6.45 ± 1.55	0.832	0.021 ± 0.0084	294.20 ± 18.24	0.771
	BC	13.66 ± 5.65	2.46 ± 0.36	0.940	0.002 ± 0.0011	335.98 ± 51.16	0.850
	PBC	28.47 ± 16.63	2.95 ± 0.74	0.834	0.004 ± 0.0030	379.28 ± 72.80	0.612
	CPBC	49.48 ± 16.73	3.4 ± 0.58	0.919	0.007 ± 0.0041	434.19 ± 54.54	0.724
Cd	CS	24.97 ± 5.77	2.86 ± 0.39	0.963	0.003 ± 0.0013	262.72 ± 27.26	0.872
	BC	16.43 ± 3.52	2.93 ± 0.36	0.953	0.003 ± 0.0007	177.84 ± 11.05	0.967
	PBC	3.26 ± 1.65	1.71 ± 0.23	0.928	0.001 ± 0.0004	368.37 ± 84.80	0.991
	CPBC	11.83 ± 1.60	2.14 ± 0.11	0.981	0.002 ± 0.0003	407.49 ± 33.80	0.974
Cr	CS	0.36 ± 0.58	1.20 ± 0.37	0.767	0.001 ± 0.0011	232.26 ± 240.53	0.794
	BC	0.39 ± 0.35	1.55 ± 0.35	0.839	0.001 ± 0.0011	54.66 ± 25.61	0.829
	PBC	0.26 ± 0.16	1.26 ± 0.16	0.950	0.001 ± 0.0004	58.77 ± 75.48	0.950
	CPBC	0.90 ± 0.76	1.51 ± 0.32	0.873	0.002 ± 0.0008	121.41 ± 33.84	0.930

**Table 4:** The Pb(II), Cd(II), and Cr(VI) sorption capacities of various modified biochars.

Biochar Raw Material	Modification Method	T (°C)	Pb Adsorption Capacity (mg·g^−1^)	Cd Adsorption Capacity (mg·g^−1^)	Cr Adsorption Capacity (mg·g^−1^)	Ref.
Corn stalk	Chitosan + K_3_PO_4_	450	434.19	407.49	121.41	This study
Hardwood	Bioapatite(femoralbones)	600	48.41	\	\	[17]
Chicken feather	H3PO4	450	\	7.84	\	[40]
Chicken feathers	H_3_PO_4_	450	55.43	\	\	[40]
Hickory wood	NaOH	600	19.10	0.98	\	[41]
Bamboo	Na_2_HPO_4_	550	\	202.66	\	[42]
Reed	Nanoscale zero-valent iron	600	38.31	39.53	23.09	[43]
Lentinula edodes	Fe_3_O_4_	600	\	\	47.62	[44]
Cherry kernel shell	Chitosan, Fe_3_O_4_	500	\	\	47.58	[45]
Sludge	HCl	600	71.52	\	\	[46]
Rice straw	Fe^3+^/Fe^2+^	600	\	42.48	\	[47]
Rice husk	Hydroxyapatite	750	110	88	\	[48]
Pine wood	Chitosan	425	134	\	\	[49]

## Data Availability

The raw data supporting the conclusions of this article will be made available by the authors on request.

## Supplementary Materials

The following supporting information can be downloaded at https://www.mdpi.com/article/10.3390/gels12080738/s1, Table S1: Kinetic models and sorption isotherms; Table S2: Fractions of HMs sorbed by materials (mg/g); Figure S1: Zeta potential of different biochar materials; Figure S2: SEM/EDX results of CPBC surface after sorption of Pb(II) (A) and Cd(II) (B); Figure S3: Electrochemical characterization of biochar before sorption. CV curves (A, B, C) and I_p_/v^1/2^ curves (D, E, F) of BC, PBC, and CPBC at different sweep rates; Figure S4: I_P_/v^1/2^ curves of BC, PBC, and CPBC after sorption of Pb(II) (A, B, C), Cd(II) (D, E, F), and Cr(VI) (G, H, I). Figure S5. Optical microscopy images of composites [38,39,73].
