# Supplementary material for: Synergistic Removal of Pb(II), Cd(II) and Cr(VI) by Chitosan-Encapsulated Phosphorus-Modified Biochar: Multi-Site Sorption and Immobilization
Source: Gels. 2026 Aug 18;12(8):738. doi: 10.3390/gels12080738 (PMC13512292; doi:10.3390/gels12080738)
Supplement: Supplementary file 1 [file gels-12-00738-s001.zip › gels-4469643-supplementary.pdf]

Supplementary Information

Synergistic Removal of Pb(II), Cd(II) and Cr(VI) by Chitosan-Encapsulated Phosphorus-Modified Biochar: Multi-Site Sorption and Immobilization

Yang Feng 1,†, Min Zhou 2,†, Jiangyan Wu 3, Lingli Li 3, Haoming Chen 1,3,* and Lingyi Tang 4,*

[1] School of Art and Design, Xijing University, Xi'an 710123, China
[2] Culinary Science and Technology Department, Jiangsu Vocational College of Tourism, Yangzhou 225000, China
[3] School of Environmental and Biological Engineering, Nanjing University of Science and Technology, Nanjing 210094, China
[4] Department of Earth and Atmospheric Sciences, University of Alberta, Edmonton, AB T6G 2E3, Canada
* Correspondence: chenhaoming@njust.edu.cn (H.C.); ltang6@ualberta.ca (L.T.)
† These authors have contributed equally to this work.

Table S1. Kinetic models and sorption isotherms

		Equation	References
Kinetic model	Pseudo-first order kinetic model (PFO)	$In(q_e - q_t) = Inq_e - k_1 t$	Deng et al. (2017)
	Pseudo-second-order kinetic model (PSO)	$\frac{t}{q_t} = \frac{1}{k_2 q_e^{2}} + \frac{t}{q_e}$	
Isothermal model	Freundlich model	$q_e = K_F C_e^{1/n}$	Chen et al. (2022)
	Langmuir model	$q_e = \frac{K_L q_m C_e}{1 + K_L C_e}$	
Unit adsorption amount (q_e)		$q_e = (C_0 - C_e) \times V/m$	Deng et al. (2017)
Removal rate (η)		$\eta = (C_0 - C_e)/C_0 \times 100\%$	Emenike et al. (2026)

Table S2. Fractions of HMs sorbed by materials (mg/g).

		Water soluble	Exchangeable	Acidic soluble	Non-bioavailable
Pb(II)	BC	30.89 ± 0.355	0.91 ± 0.0214	71.90 ± 0.467	56.30 ± 1.042
	CS	124.70 ± 3.180	0.18 ± 0.00081	127.16 ± 1.717	3.96 ± 0.1089
	PBC	38.46 ± 0.365	2.52 ± 0.0542	112.86 ± 0.395	71.16 ± 1.174
	CPBC	29.99 ± 0.885	0.82 ± 0.00615	91.69 ± 1.330	161.27 ± 3.629
Cd(II)	BC	24.82 ± 0.137	10.54 ± 0.279	31.44 ± 0.393	17.05 ± 0.332
	CS	76.54 ± 0.191	0.54 ± 0.0132	75.06 ± 0.638	0.43 ± 0.00667
	PBC	4.85 ± 0.138	15.48 ± 0.163	69.50 ± 1.425	28.36 ± 0.142
	CPBC	26.04 ± 0.456	0.59 ± 0.00177	87.94 ± 2.023	49.07 ± 0.540
Cr(VI)	BC	3.10 ± 0.0837	0.34 ± 0.00238	0 ± 0	9.66 ± 0.135
	CS	0.09 ± 0.00270	0.37 ± 0.00222	22.92 ± 0.435	0.21 ± 0.00252
	PBC	3.97 ± 0.103	0.06 ± 0.00024	0.08 ± 0.00160	7.73 ± 0.0618
	CPBC	0.73 ± 0.0175	0.08 ± 0.00104	8.83 ± 0.194	2.85 ± 0.0257

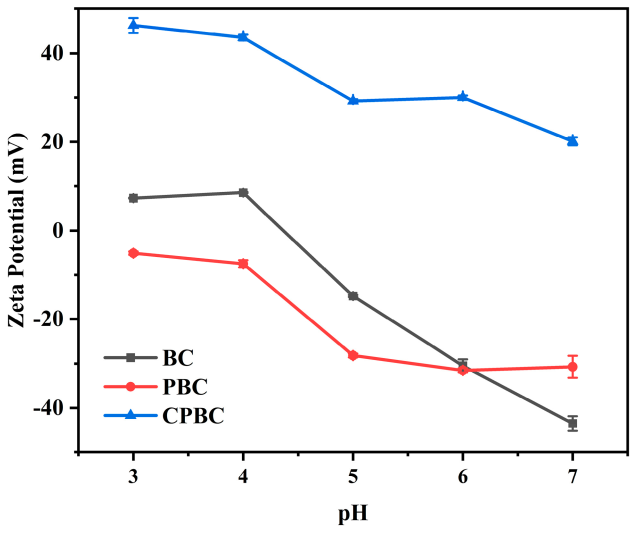

Figure S1. Zeta potential of different biochar materials

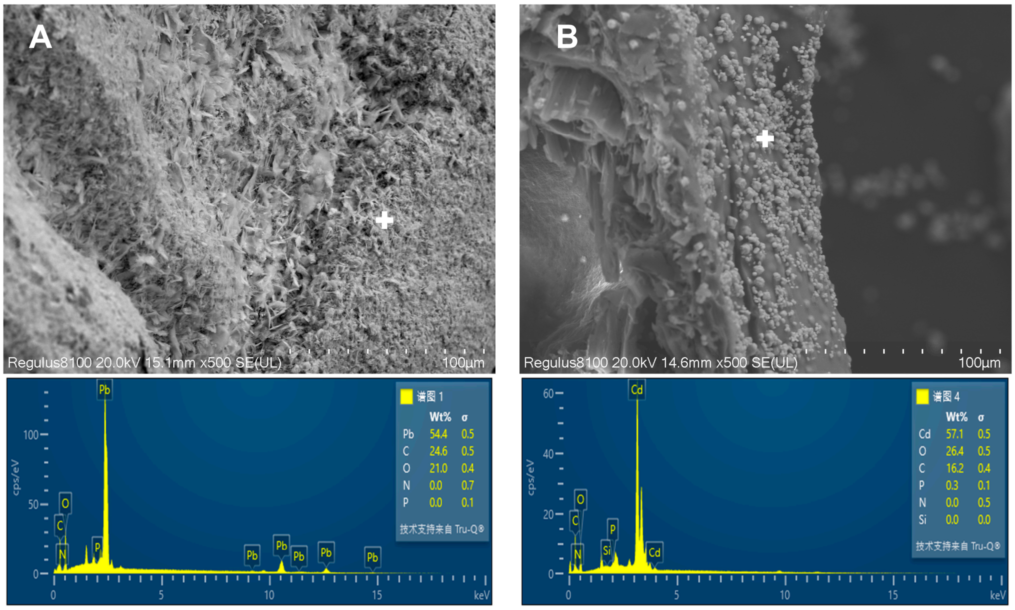

Figure S2. SEM/EDX results of CPBC surface after sorption of Pb(II) (A) and Cd(II) (B).

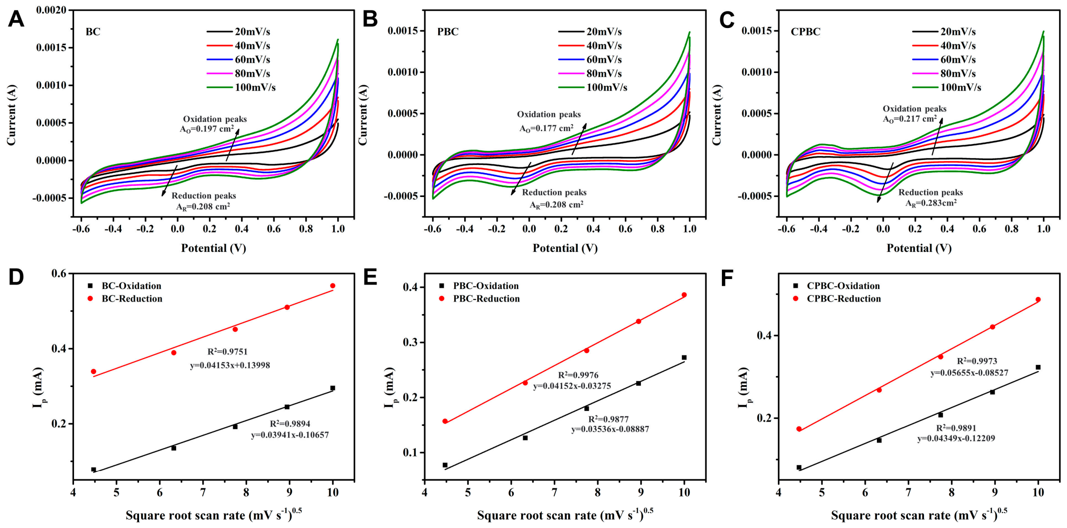

Figure S3. Electrochemical characterization of biochar before sorption. CV curves (A, B, C) and $I_p/v^{1/2}$ curves (D, E, F) of BC, PBC and CPBC at different sweep rates.

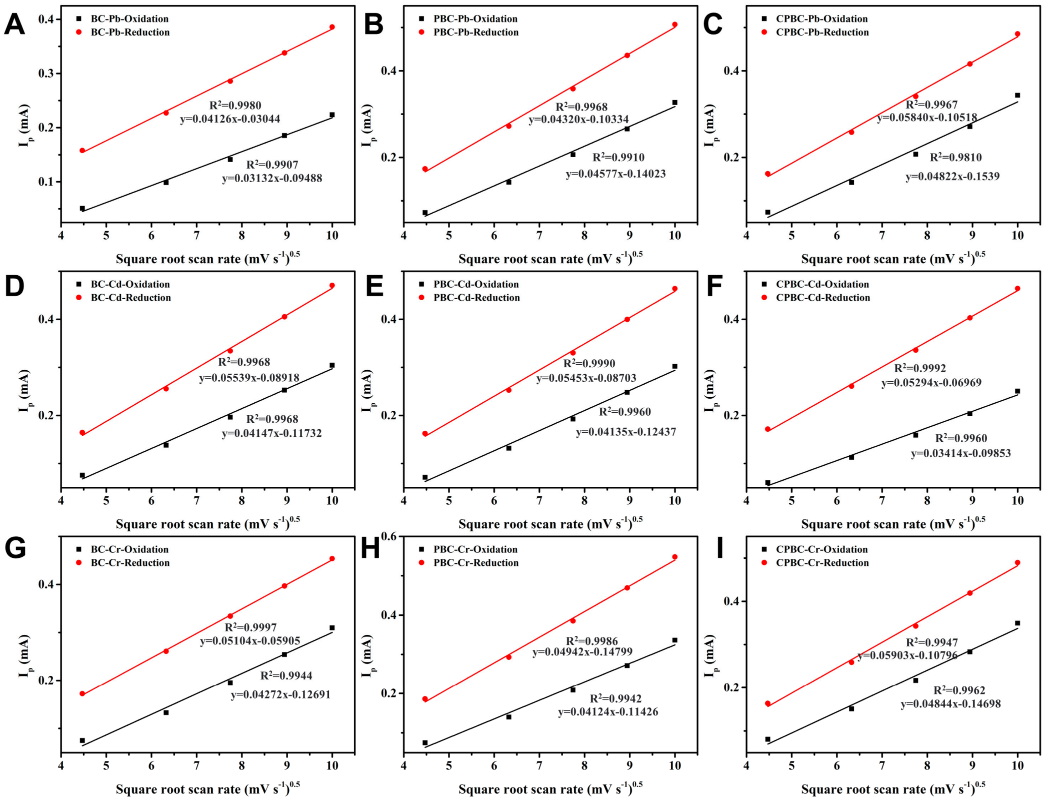

Figure S4. $I_P/v^{1/2}$ curves of BC, PBC and CPBC after sorption of Pb(II) (A, B, C), Cd(II) (D, E, F) and Cr(VI) (G, H, I).

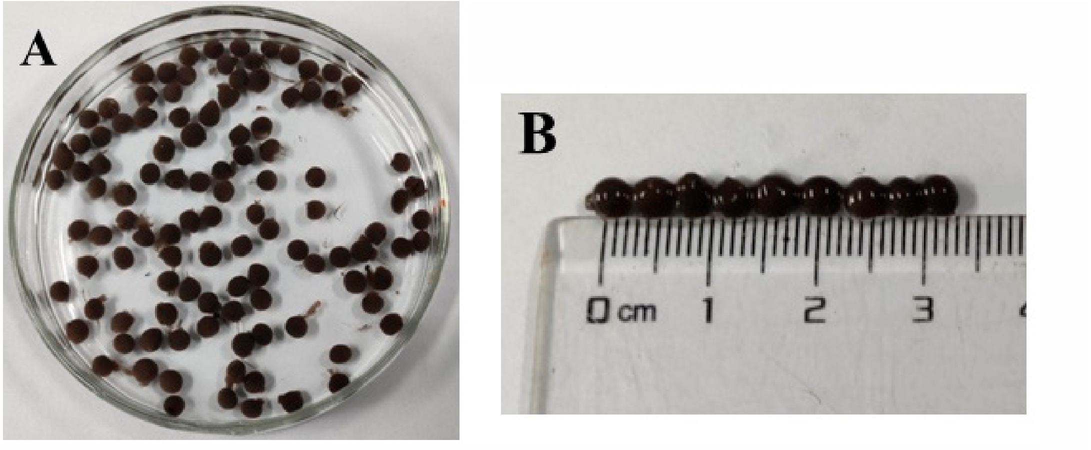

Figure S5. Optical microscopy images of composites.

References:

Deng J, Liu Y, Liu S, et al. Competitive adsorption of Pb(Ⅱ), Cd(Ⅱ) and Cu(Ⅱ) onto chitosan-pyromellitic dianhydride modified biochar[J]. Journal of Colloid and Interface Science, 2017, 506: 355-64.

Chen X, Hossain M F, Duan C, et al. Isotherm models for adsorption of heavy metals from water - A review[J]. Chemosphere, 2022, 307(Pt 1): 135545.

Emenike E C, Okoro H K, Al-Senani G M, et al. Adsorptive removal of Cr(VI) and Pb(II) using biochar from biomass and plastic waste: characterization and performance evaluation[J]. Biomass and Bioenergy, 2026, 207: 108722.
